# Organic nitrogen enhances nitrogen nutrition and early growth of *Pinus sylvestris* seedlings

**DOI:** 10.1093/treephys/tpab127

**Published:** 2021-09-28

**Authors:** Hyungwoo Lim, Sandra Jämtgård, Ram Oren, Linda Gruffman, Sabine Kunz, Torgny Näsholm

**Affiliations:** Department of Forest Ecology & Management, Swedish University of Agricultural Sciences (SLU), Skogsmarksgränd 17, SE-901 83 Umeå, Sweden; Department of Forest Ecology & Management, Swedish University of Agricultural Sciences (SLU), Skogsmarksgränd 17, SE-901 83 Umeå, Sweden; Department of Forest Genetics and Plant Physiology, SLU, Skogsmarksgränd 17, SE-901 83 Umeå, Sweden; Nicholas School of the Environment, Duke University, Durham, Grainger Hall, 9 Circuit Drive, NC 27708-0328, USA; Department of Forest Science, University of Helsinki, Latokartanonkaari 7, FI-00014 Helsinki, Finland; Department of Forest Ecology & Management, Swedish University of Agricultural Sciences (SLU), Skogsmarksgränd 17, SE-901 83 Umeå, Sweden; Department of Forest Genetics and Plant Physiology, SLU, Skogsmarksgränd 17, SE-901 83 Umeå, Sweden; Department of Forest Ecology & Management, Swedish University of Agricultural Sciences (SLU), Skogsmarksgränd 17, SE-901 83 Umeå, Sweden; Department of Forest Genetics and Plant Physiology, SLU, Skogsmarksgränd 17, SE-901 83 Umeå, Sweden

**Keywords:** amino acids, arginine, microdialysis, nitrate, soil N availability, water-use efficiency

## Abstract

Boreal trees are capable of taking up organic nitrogen (N) as effectively as inorganic N. Depending on the abundance of soil N forms, plants may adjust physiological and morphological traits to optimize N uptake. However, the link between these traits and N uptake in response to soil N sources is poorly understood. We examined *Pinus sylvestris* L. seedlings’ biomass growth and allocation, transpiration and N uptake in response to additions of organic N (the amino acid arginine) or inorganic N (ammonium nitrate). We also monitored in situ soil N fluxes in the pots following an addition of N, using a microdialysis system. Supplying organic N resulted in a stable soil N flux, whereas the inorganic N resulted in a sharp increase of nitrate flux followed by a rapid decline, demonstrating a fluctuating N supply and a risk for loss of nitrate from the growth medium. Seedlings supplied with organic N achieved a greater biomass with a higher N content, thus reaching a higher N recovery compared with those supplied inorganic N. In spite of a higher N concentration in organic N seedlings, root-to-shoot ratio and transpiration per unit leaf area were similar to those of inorganic N seedlings. We conclude that enhanced seedlings’ nutrition and growth under the organic N source may be attributed to a stable supply of N, owing to a strong retention rate in the soil medium.

## Introduction

Growth of boreal trees is generally limited by a low soil nitrogen (N) availability, which is believed to be caused by low mineralization rates (Ingestad 1977, Tamm 1991, Linder 1995, McMurtrie et al. 2001, LeBauer and Treseder 2008). Hence, the addition of mineral N (inorganic N; ammonium nitrate) has long been practiced to improve nutrition and growth of boreal trees in the field, but also when cultivating conifer seedlings in nurseries (Ingestad 1977, Tamm 1991, Linder 1995, Lim et al. 2017). Recent research has demonstrated that plants are highly capable of absorbing organic N, such as peptides and amino acids, thus circumventing the need for mineralization (Kielland 1994, Lipson and Monson 1998, Näsholm et al. 1998, Schmidt and Stewart 1999, Persson and Näsholm 2001, Bueno et al. 2019). Amino acid transporters involved in root uptake of amino acids have been identified in plants, providing a mechanism for acquisition of amino acids regardless of mycorrhizal colonization state (Hirner et al. 2006, Lee et al. 2007, Svennerstam et al. 2007, 2008, Näsholm et al. 2009, Ganeteg et al. 2017). Amino acids, which are among the most widespread forms of soil organic N, constitute a large share of available N fluxes in boreal soils (Inselsbacher and Näsholm 2012), and are efficiently taken up by boreal tree species (Näsholm et al. 1998, Nordin et al. 2001, Gruffman et al. 2012, Hedwall et al. 2017). These findings have led to the development of an organic N fertilizer based on amino acids, e.g., arginine (Öhlund and Näsholm 2001).

In response to chemical forms of soil N, plants may adjust physiological and morphological traits to optimize N uptake. For example, nitrate, an extremely mobile anion in the soil matrix, can effectively be delivered to the root surface via transpiration-driven mass flow (Oyewole et al. 2014, McMurtrie and Näsholm 2018, Henriksson et al. 2021). In this case, plants may increase hydraulic conductivity and transpiration to take up the mobile N source (Cramer et al. 2008, Gorska et al. 2008, Matimati et al. 2014, Graciano et al. 2016), and, in the long-term, allocate more biomass to shoot relative to roots, enhancing transpiration capacity (Cambui et al. 2011). In contrast, arginine, a positively charged amino acid, is tightly bound to soil particles, making its diffusion rate in the soil matrix extremely low (Inselsbacher and Näsholm 2012, Oyewole et al. 2014, 2016). Thus, it is reasonable to expect that, in an arginine-dominated soil matrix, plants should allocate more biomass to root growth, increasing root surface area and soil exploration of immobile N.

It is well documented that high amounts of ammonium-N in the soil may cause soil acidification and limit uptake of other cations (e.g., potassium, magnesium and calcium) (Meyer et al. 1988), leading to nutrient imbalance (Boxman and Roelofs 1988, Oren et al. 1988, Osonubi et al. 1988, Rollwagen and Zasoski 1988, Griffin et al. 1995). To avoid such issues, nutrition of conifer seedlings is generally managed with additions of ammonium nitrate. However, this leads to poor N recovery because conifer seedlings prefer ammonium over nitrate (Marschner et al. 1991, Knoepp et al. 1993, Kronzucker et al. 1997). For example, Kamminga-van wijk and Prins (1993) showed that the presence of ammonium almost entirely inhibits uptake of nitrate throughout the range of tested N concentrations (10–1000 μM). Moreover, the retention of nitrate is low in most growth media. This, combined with low uptake rates, results in N leaching and thus poor recovery of added N (Öhlund and Näsholm 2002). Supplying seedlings with organic N fertilizer has been shown to mitigate these issues. Uptake of the amino acids arginine or glycine is not inhibited by the presence of ammonium (Gruffman et al. 2014). Furthermore, when plant internal N concentration is high, uptake of N generally decreases for ammonium and nitrate, but not for arginine or glycine (Öhlund and Näsholm 2001, 2004, Persson and Näsholm 2002, Gruffman et al. 2014), potentially because of the reduced dependency on current photosynthates for the uptake and metabolism of amino acids (Gruffman et al. 2013, Franklin et al. 2017). Basic amino acids, in particular arginine, were also shown to contribute a significant share of available N to tussock tundra plants (Homyak et al. 2021), suggesting a broad utilization of this source of N by various plants.

Nitrate can quickly be lost after an addition, through leaching, microbial immobilization or denitrification, whereas arginine, for example, attaches to soil particles and may be subsequently mineralized, slowly releasing inorganic N (Öhlund and Näsholm 2001, 2002). Monitoring temporal changes in N fluxes, as an indication of its availability to plant roots, in intact soil without disturbing the soil–root system is challenging. We overcome this challenge by using a microdialysis technique that allows monitoring of soil N fluxes in situ with minimal disturbance, using induced diffusive fluxes from soil solutions (Inselsbacher et al. 2011, Inselsbacher and Näsholm 2011, Oyewole et al. 2014, Buckley et al. 2019).

The aim of the present study was to examine soil N fluxes and seedling performance under inorganic N (ammonium nitrate) and organic N (the amino acid arginine) nutrition. The aim was also to investigate physiological and morphological traits that are associated with root uptake of different N forms. We studied N uptake, biomass growth and allocation, root morphology and transpiration of *Pinus sylvestris* L. seedlings, as well as diffusive soil N fluxes using microdialysis. Seedlings’ N stock was used as a proxy of N uptake. We also assessed foliar ^13^C natural abundance (expressed as δ^13^C) to infer the effect of N source on water-use efficiency, the amount of carbon assimilation per unit transpiration (Farquhar et al. 1982).

We hypothesized that

(i) the organic fertilizer would provide a slower and more stable N supply of N than inorganic fertilizer (H1),
(ii) seedlings supplied with the organic fertilizer would accumulate a greater N stock than seedlings supplied with the inorganic fertilizer (H2) and
(iii) at similar N status, seedlings supplied with the organic fertilizer would produce greater biomass (H3a), and display a higher root-to-shoot ratio (H3b), a higher root area per unit root mass (H3c) and lower transpiration rate (H3d).

## Materials and methods

### Plant material and growth conditions

*Pinus sylvestris* seeds (~5 g per 1000 seeds) were sown in 70 g of soil (unfertilized dolomite peat, vermiculite and sand, 1:1:1) in 1.3 dl pots (one seed per pot), and grown in a greenhouse at the Swedish University of Agriculture Sciences, Umeå. Two weeks after sowing, 64 of the seedlings were randomly selected, and eight seedlings were assigned to each of eight blocks; seedlings were rotated twice a week within a block throughout the experiment in order to minimize variations within block. Conditions in the greenhouse were day/night 16/8 h, 20/18 °C; 70–80% relative humidity, and 150 μmol m^−2^ s^−1^ average photosynthetic photon flux density.

Two N sources were used: an arginine-based organic fertilizer (g l^−1^; 65 arginine-N, 11 P, 45 K, 4 Mg, 9 S, 0.22 B, 0.03 Cu, 1.1 Fe, 0.5 Mn, 0.04 Mo, 0.16 Zn; arGrow®, Arevo AB, Umeå, Sweden) and ammonium nitrate-based inorganic fertilizer (g l^−1^; 51.6 NO_3_^—^N, 32.4 NH_4_^+^-N, 12 P, 56.4 K, 7.2 Mg, 9.6 S, 0.1 B, 0.02 Cu, 0.8 Fe, 0.5 Mn, 0.02 Mo, 0.12 Zn; Rika-S, Weibulls, Hammenhög, Sweden). The N:P:K ratio was 100:17:69 for the organic fertilizer and 100:14:67 for the inorganic fertilizer. Fertilizer stock solution (5 mole N l^−1^; 5 mM N) was prepared for each fertilizer, the N concentration of which was determined using a Shimadzu analyzer (TNM-1, Shimadzu, Kyoto, Japan), and was portioned in to 50 ml, and kept frozen to preserve the intact form of N. In each block, four seedlings were grouped and supplied with the organic fertilizer and four with the inorganic fertilizer. Seedlings were fertilized two to three times a week with 20 ml of diluted fertilizer solution (5 mM N; 1.4 mg N) from the fertilizer stock solutions. By the end of the experiment, each seeding had received a total 33.6 mg N (10 weeks; 24 occasions).

### Transpiration and water uptake rates

Ten weeks after the fertilization was initiated, three seedlings from each N-treatment were selected from six of the blocks and assessed for transpiration (or uptake of soil water) during a 46-h period. Thereafter, the seedlings were harvested for biomass measurements and chemical analyses. A total of 16 seedlings (blocks 7 and 8) were used to monitor dynamics of soil N fluxes (arginine, ammonium and nitrate) following fertilizer applications, using the soil microdialysis technique (Inselsbacher et al. 2011).

Seedlings’ transpiration (and soil water uptake) was estimated based on a mass-balance approach. The soil in the pots was sealed with plastic film to avoid evaporation. Beginning 1 h after fertilizer solutions were applied, seedling pots were weighed at predetermined intervals (1, 4, 9, 12, 24, 32, 46 h). The difference in weight of a pot between two time points was considered transpired water, which was then normalized by leaf area and time, expressed as transpiration rate (mmol H_2_O m^−2^ leaf area s^−1^), and root area and time, expressed as soil water uptake rate (mmol H_2_O m^−2^ root area s^−1^), assuming neither root area nor leaf area changed over the 46 h. After weighing all pots for transpiration estimation, all seedlings were harvested.

### Soil nitrogen flux monitoring using soil microdialysis

The following day (i.e., 3 days after the previous fertilization event) induced diffusive flux rates of N in the soil were monitored in the pots of seedlings from blocks 7 and 8 with soil microdialysis (Inselsbacher et al. 2011). In each of the 16 pots, a microdialysis probe was inserted into the growth medium between the stem and an edge of the pot, with a polyarylethersulphone membrane (30 mm × 0.5 mm, surface area of 0.4732 cm^2^ with a 20 kDa molecular weight cutoff). High-purity distilled water (MilliQ, Merck Millipore Corp., Billerica, MA, USA) was perfused through the probes at a flow rate of 5 μl min^−1^ for 30 min, with syringe infusion pumps (CMA 4004) equipped with gas-tight glass syringes (2.5 ml; CMA microdialysis AB, Solna, Sweden), with each syringe connected to a microdialysis probe. Samples from the probes (dialysates) were collected in a 1.5 ml tube and kept on ice. Sampling was carried out at seven time points; 1 h before applying fertilizer solutions, and thereafter at the selected time points after fertilizer application (1, 2, 7, 22, 30, 48 h). As before, four of the seedlings from each block received the arginine-based organic fertilizer solution and the other four seedlings from each block received the ammonium nitrate-based inorganic fertilizer solution. Collected dialysates were frozen until analyzed, to preserve the N forms. Dialysates were analyzed for ammonium, nitrate and arginine as described by Inselsbacher et al. (2011). Briefly, nitrate in the dialysates was analyzed cholorimetrically based on the method described by Miranda et al. (2001) using the vanadium (III) chloride (VCl_3_) and the Griess method. Ammonium and arginine were analyzed by reversed phase liquid chromatography using a Waters Ultra High Performance system with a Waters Tunable UV detector. Samples were derivatized with a Waters AccQ-Tag™ Ultra Derivatization kit for amino acid analysis.

Dialysate concentration represents induced diffusive flux rates of the solutes and thus was expressed as N flux rate (nmol N m^−2^ s^−1^).

The N flux rate (nmol N m^−2^ s^−1^) = N concentration in the final dialysate (nmol N μl^−1^) × the volume of the collected dialysate (μl)/[the surface area of the membrane (4.732 × 10^−5^ m^2^) × time (s)].

Mean diffusive N flux rate between time points was then multiplied by the time period to estimate total cumulative N flux over the monitoring period.

### Analysis of harvested seedlings

Aboveground parts were cut at the first root branching, and put in plastic bags. Roots were carefully rinsed with water to remove soil particles, and covered with wet paper tissue and put in plastic bags. Aboveground parts were separated into foliage and stem with branches. Fresh foliage was scanned using a flatbed scanner (Epson 1600), and projected leaf area was estimated using ImageJ 1.50e software (Rasband WS, National Institutes of Health, Bethesda, MD, USA). Thereafter, foliage was oven-dried (65 °C for 72 h). Root surface area was estimated using WinRHIZO (Regent Instruments Inc., Sainte-Foy, QC, Canada) equipped with a scanner (STD4800), and then roots were oven-dried (65 °C for 72 h). Samples of stem and branches were also oven-dried. Foliage and roots samples were analyzed for carbon and N, and ^13^C and ^15^N using a Flash EA 2000 (Thermo Fisher Scientific).

Foliage and root N stocks were estimated by multiplying the dry mass by the corresponding N concentration for each seedling. The N content of stem was estimated using relative N content ratio of stem to foliage, which was taken from a similar experimental setting (Gruffman 2013). By multiplying dry mass of stem by the estimated N concentration, and summing with the N content of leaves and roots, total N stock of each seedling was estimated.

### Statistical analyses

The effects of N source on seedlings’ traits were determined using a linear mixed model to account for randomized complete block design.

*Y_ijk_ = a + b_1_ × T_j_ + b_2_ × N_ijk_ + b_3_ × TN_ijk_ + ε_k_ + ε_ijk_* (1)

where *Y_ijk_* is the response variable in the *i*th seedling (i = 1–3) under *j*th treatment *T* (*j* = organic or inorganic fertilizer) with *k*th block (*k* = 1–6). *N* is foliar N concentration, *TN* is interaction term between treatment *T* and *N*, *a* is intercept, *b_s_* are coefficients to be estimated, *ε_k_* is the random residuals associated with block and *ε_ijk_* is the final residuals. The effect of N source on transpiration was also examined using Eq. (1), separated for each sampling point. Excepting shoot biomass, coefficients of *N* and *TN* were not different from 0 (*P* > 0.05), and thus, the variables were removed in the analysis of other response variables. Means and their uncertainties, and the relative effect between the two treatments were estimated based on the model outcomes. For shoot biomass, which was responsive to foliar N concentration with interaction with treatment, the estimates were thus normalized by the mean of foliar N concentration for each treatment (Table S2 available as Supplementary data at *Tree Physiology* Online). The effect of treatment on soil N flux was assessed using a two-sample t-test for each measurement point.

Statistical analyses were performed using R (v. 3.2.2): the *lme* function in the nlme package (Pinheiro et al. 2020) was used for the mixed model (Eq. (1)); *lsmeans* and *cld* functions in the lsmeans package (Lenth 2016) were used to compute mean and standard error of the estimates.

## Results

### Soil nitrogen fluxes

**Figure 1.**
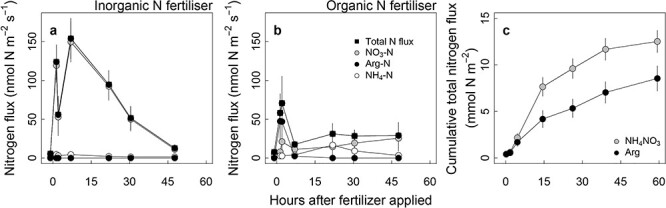
Soil N fluxes from the growth medium of seedling-pots supplied with 5 mM N of (a) the inorganic N (ammonium nitrate) or (b) organic (arginine) fertilizer. (c) The cumulative total N flux over the monitoring period. Fertilizers were applied at hour 0, and samples were collected using soil microdialysis. Error bars are standard errors (*n* = 8). Detailed values and statistical results are given in Table S1 available as Supplementary data at *Tree Physiology* Online.

At the start of the induced diffusive flux rate measurements (3 days after the previous fertilizer application, at time −1 h), the diffusive flux of both N sources was ~ 6 nmol N m^−2^ s^−1^ (*P* = 0.368; Figure 1, Table S1 available as Supplementary data at *Tree Physiology* Online). Immediately after the fertilizers were applied, soil N flux rates increased. In pots supplied with the inorganic fertilizer, soil nitrate flux rate increased sharply and then linearly declined over the following 41 h (Figure 1a). The application of the organic fertilizer increased N fluxes to about a third of the flux from the inorganic N application, but following some decline, the flux remained relatively stable for the rest of the monitoring period (Figure 1b). The organic fertilizer resulted in increased flux rates of arginine, ammonium and nitrate, whereas the inorganic fertilizer mainly resulted in nitrate flux. Over the 3 days of the N flux monitoring, the inorganic N fertilizer resulted in a higher cumulative N flux than the organic fertilizer did (12.52 ± 1.19 *vs* 8.54 ± 1.35, *P* = 0.045; Figure 1c).

### Response of seedlings to nitrogen source

**Figure 2.**
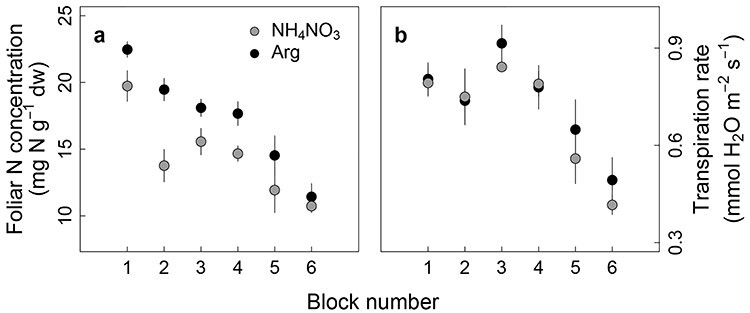
Cross-block variation of (a) foliar N concentration and (b) transpiration rate. Error bars are standard errors (*n* = 3 within a block).

**Figure 3.**
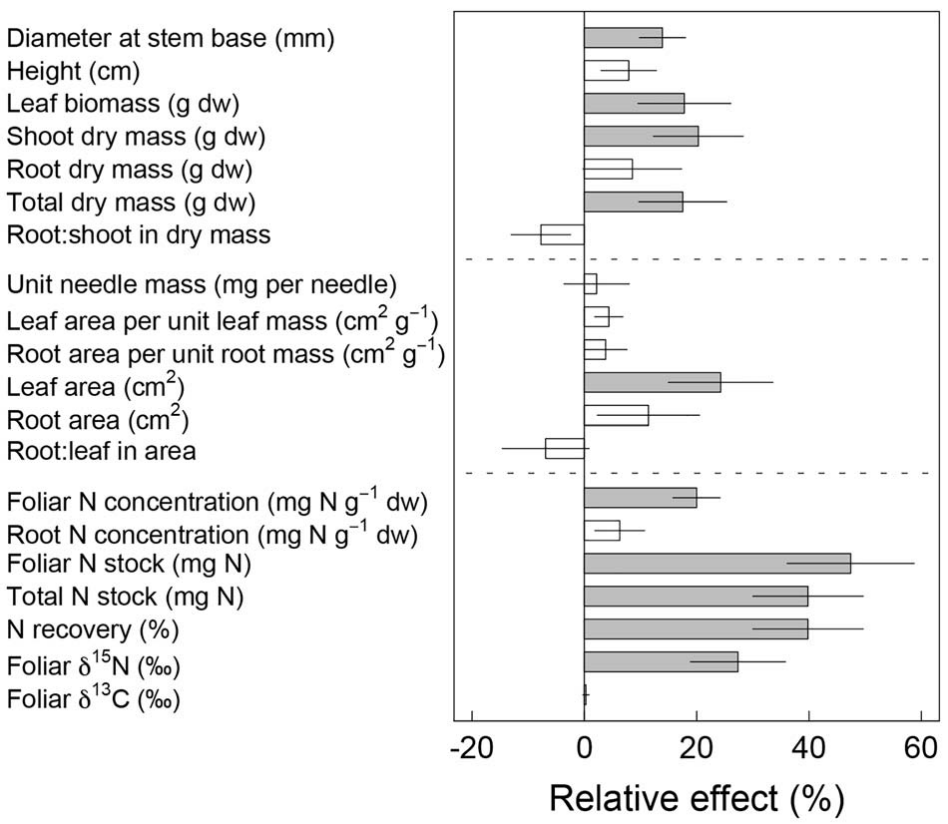
The effect on traits of *Pinus sylvestris* seedlings of arginine-based organic fertilizer relative to ammonium nitrate-based inorganic fertilizer. The effects of N source were determined and quantified using a linear mixed model (Eq. (1)), and the relative effect was estimated using the model outcomes, [(organic – inorganic)/inorganic] ± standard error (*n* = 6 blocks). Gray bars indicate that the effect is different from 0 (*P* < 0.05). Detailed values and statistical results are given in Table S2 available as Supplementary data at *Tree Physiology* Online.

**Figure 4.**
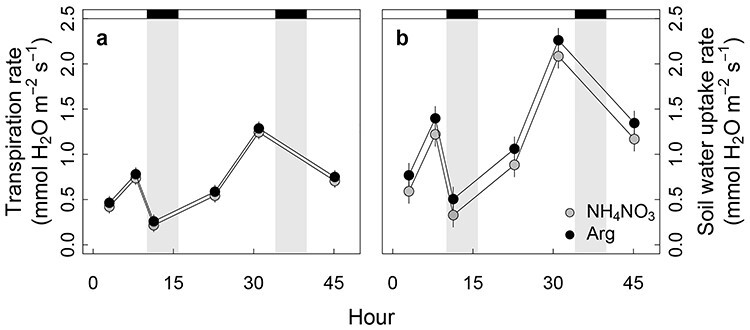
(a) Transpiration rate per unit of leaf area and (b) soil water uptake rate per unit of root area. Gray circles, inorganic (ammonium nitrate) fertilizer; dark circles, organic (arginine) fertilizer. The fertilizer solutions were applied at time 0. Gray areas are dark periods; error bars are standard errors (*n* = 6 associated with block).

Significant cross-block variations masked the fixed effects, treatment, foliar N concentration and their interaction (Figure 2). The use of a linear mixed model (Eq. (1)) that constrains the random block effect therefore facilitated an assessment of fixed effects on response variables (Figures 3 and 4).

The organic fertilizer increased seedlings’ growth and N nutrition (Figure 3, Table S2 available as Supplementary data at *Tree Physiology* Online). Stem diameter was 13.9 ± 4.1% larger for seedlings supplied with the organic fertilizer, whereas seedlings’ height did not differ between the two treatments (*P* = 0.120). Seedlings supplied with arginine-based fertilizer had greater shoot biomass (20.3 ± 8.0%; *P* = 0.017), leaf biomass (17.8 ± 8.3%; *P* = 0.040) and leaf area (24.3 ± 9.3%; *P* = 0.015), compared with seedlings supplied with ammonium nitrate-based fertilizer. However, there was no significant effect of N source on unit needle mass (*P* = 0.710) or leaf area per unit leaf mass (*P* = 0.095). Root biomass, root area or root area per unit root mass were not affected by the treatment (*P* ≥ 0.221). The N source did not affect allocation patterns, either for root-to-shoot biomass ratio (*P* = 0.155) or for root-to-foliage area ratio (*P* = 0.381). The organic fertilizer resulted in a 20.0% ± 4.2% higher foliar N concentration and a 47.4 ± 11.3% greater foliar N stock, compared with seedlings supplied with the inorganic fertilizer. Foliar δ^13^C values were not different between the two N source treatments (−30.8 ± 0.1‰; *P* = 0.614). The N recovery of applied N was 39.8 ± 9.8% higher for the organic N treatment compared with the inorganic N treatment.

Transpiration rates per unit leaf area (Figure 4a) and rate of soil water uptake (transpiration per unit root surface area; Figure 4b) followed a diurnal pattern, decreasing during the dark period and increasing during the light period, but were not affected by the treatment (*P* = 0.412 for transpiration rate and *P* = 0.137 for water uptake rate).

### Relationships between seedlings’ traits

**Figure 5.**
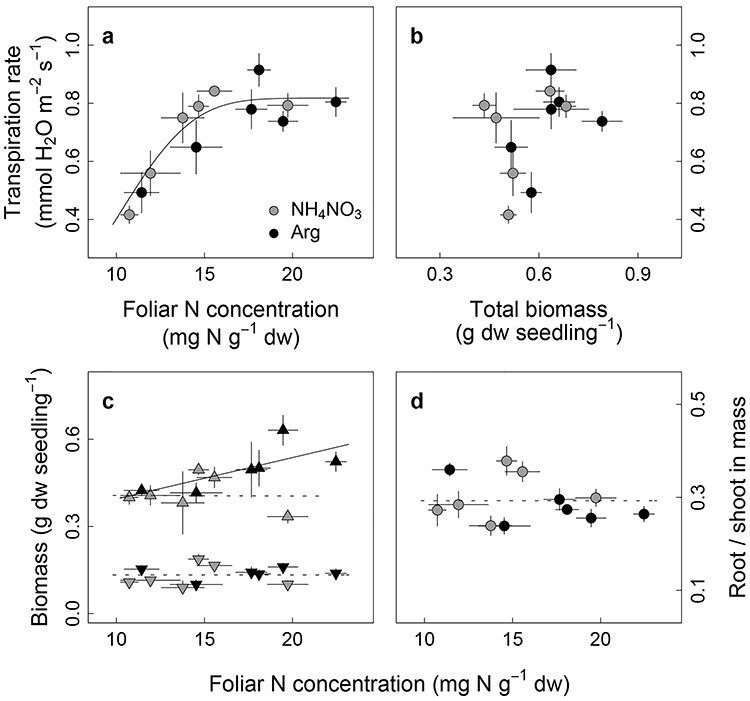
Relationships of seedlings’ traits (a) between transpiration rate and foliar N concentration (mg N per g dry weight), (b) transpiration rate and total biomass (shoot + roots), (c) shoot biomass (up-pointing triangle) or root biomass (down-pointing), and foliar N concentration, and (d) root-to-shoot ratio and foliar N concentration. Error bars are standard error (*n* = 3 within a block).

Transpiration rate was positively related to foliar N concentration but no effect of N source was detected on the relationship (Figure 5a). A derived relationship using a Weibull function (*y* = *a* × Exp(−(*x*/*b*)^*c*^), to allow for a response saturation, resulted in an asymptote, *a*, of 0.82 mmol H_2_O m^−2^ leaf area s^−1^, an inflection point, *b*, of 11.20 mg N g^−1^ dry weight and growth rate, *c*, of 3.44 (R^2^ = 0.71; Figure 5a). There was no correlation between transpiration rate and total biomass of seedlings (shoot + root; Figure 5b). Shoot biomass increased with foliar N concentration of the organic fertilizer seedlings (*P* = 0.048), but it was unrelated to foliar N of the inorganic seedlings (*P* = 0.280; Figure 5c). Neither root biomass (*P* = 0.577 for the coefficient of a fixed variable, *N*, in Eq. (1)) nor root-to-shoot ratio (*P* = 0.277) correlated with foliar N concentration (Figure 5d).

## Discussion

We examined the effects of N source, organic or inorganic forms, on in situ availability of soil N, and the growth response and associated functional traits of *P. sylvestris* seedlings. This approach allowed an assessment of the impacts of organic N source on seedlings’ N economy, physiological and morphological responses, and potential mechanisms associated with these responses.

We found that seedlings supplied with the organic fertilizer (the amino acid arginine) had higher N concentration and larger biomass (supporting H3a), reflecting a substantially higher N recovery of the supplied fertilizer (supporting H2), compared with seedlings supplied with the inorganic fertilizer (ammonium nitrate; Figure 3, Table S2 available as Supplementary data at *Tree Physiology* Online). Based on classical diagnostic interpretations (Timmer and Stone 1978), the higher foliar N concentration and its content, but similar unit needle weight of the organic seedlings, reflect better N uptake and storage without toxicity, compared with the inorganic seedlings (Figure S1 available as Supplementary data at *Tree Physiology* Online). Application of the organic fertilizer resulted in low but stable supply rates of mostly arginine, but also ammonium and nitrate, with no clear sign of abating by the end of the study period (Figure 1). In contrast, the inorganic fertilizer resulted in high nitrate availability immediately following application, progressively increasing during the first 7 h and thereafter decreasing to near background levels within 48 h. These results provide support for H1, that organic N provides a slower and more stable N supply.

The difference in the initial rise of available nitrate-N from the inorganic fertilizer versus available N from the organic fertilizer treatment may relate in part to the different charges of the molecules. Arginine is positively charged at the soil solution pH used in this experiment, and therefore binds to the negatively charged surfaces of the growth substrate, and potentially to root surfaces. In contrast, nitrate is negatively charged and therefore remains in soil solution (Inselsbacher et al. 2011). The difference in availability may also be due to *P. sylvestris'* preferential uptake of arginine compared with nitrate (Öhlund and Näsholm 2001, Gruffman et al. 2014). In our study, a high concentration of nitrate-N in the soil solution followed the fertilization event. According to Öhlund and Näsholm (2002), this could have led to high leaching losses of N through soil profile, as reflected in a decline of nitrate flux. Even though we observed that the total N availability was relatively stable over time with the addition of the organic fertilizer, the induced diffusive fluxes of arginine disappeared 7 h after the addition, while ammonium and nitrate availability increased, most likely related to microbial mineralization of arginine. Combined, these fluxes were smaller than the corresponding nitrate fluxes in the inorganic fertilizer treatment. This is consistent with the findings of Öhlund and Näsholm (2002), where a large portion of intact arginine (56%) was taken up by *P. sylvestris* seedlings within the first hour, followed by uptake of mostly mineralized N, suggesting that the reduction of arginine-N flux rates resulted from depletion caused by root uptake and mineralization. In that study, ~ 70% of the added nitrate was lost, while the loss of ammonium was only 37%. In the present study, the inorganic fertilizer resulted in very low ammonium flux rates. This may be explained by both the preferential uptake of this N form (over nitrate) by roots, and its ability, similarly to arginine, to bind to the negatively charged surfaces of the growth substrate (Inselsbacher et al. 2011, Gruffman et al. 2014).

The supposition that the lower flux of arginine, or nitrate in the arginine treatment, may have resulted from binding to negatively charged soil surfaces is further supported by results from addition of the non-charged amino acid glutamine to soil (Ganeteg et al. 2017). In that case, more glutamine in solution permitted higher mineralization, resulting in more than twice the nitrate levels as observed with arginine. The lower magnitude of available N in the organic fertilizer treatment might have resulted in a low rate of N acquisition. However, the experiment revealed the opposite outcome—the recovery of N by the seedlings was greater with the organic fertilizer than the inorganic fertilizer (Figure 3). This is consistent with the classical results of Ingestad (1962, 1977), whereby the best recovery of a nutrient through root uptake is when the supply rate matches the demand of seedlings to support growth. It is quite likely that the high-amplitude pulses of nitrate-N following each fertilization event provided far more N than the seedlings can take up or use, followed by a period of insufficient supply (Figure 1a; Brackin et al. 2015). In contrast, the organic N source provided a steady supply of N (Figure 1b).

Roots take up N that is delivered to their surface via mass flow, which is mainly driven by transpiration, and diffusive flux, which is driven by the N concentration gradient resulting from active uptake of N at the root surface. Despite this, the relationship between transpiration and foliar N concentration was unaffected by N source (Figure 5a), and neither leaf nor root morphology was affected by N source (Figure 3). Therefore, we conclude that N form did not affect the uptake mechanism associated with mass flow, nor did it shift the hydraulic allometry of the seedlings, thus refuting H3b–d. (Figure 3). Instead, higher N recovery and higher growth with the organic fertilizer may, at least partly, result from the observed stable supply of N via diffusive fluxes, owing to strong retention rates of arginine in the growth substrate and less N leaching below the reach of the developing root system.

**Figure 6.**
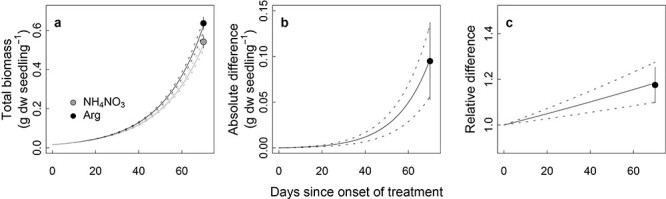
Growth simulation using an exponential growth model (current biomass = initial biomass × *e*^*r* × time^) during the treatment period after the germination. (a) Total biomass development, (b) difference of biomass between the treatments and (c) relative difference. Initial biomass was set to 15.84 mg as an expected seed weight following the growth model at the onset of the fertilization treatment (23 days after the germination, an initial seed weight of 5 mg). Error bars and dotted lines are standard error of estimates associated with block (*n* = 6).

The greater biomass production of seedlings supplied with organic N resulted from increased shoot biomass, with a positive correlation with foliar N concentration (Figure 5c). Surprisingly, and in contrast to published studies (Ågren and Franklin 2003, Ågren and Ingestad 1987), such correlation was not observed for seedlings supplied with inorganic N. The results show that foliar δ^13^C and transpiration rate were unaffected by the N source (Figures 3 and 4), and that light-normalized photosynthetic rate, stomatal conductance per unit leaf area and water-use efficiencies were similar (via gas-exchange measurements, Figure S2 available as Supplementary data at *Tree Physiology* Online). Thus, higher foliar N content of the organic seedlings did not lead to higher carbon assimilation per unit transpiration or per unit leaf area in the given light condition. This is consistent with findings from studies showing that, once photosynthetic and growth requirements are met, uptake in excess of demand may accumulate as nontoxic foliar N storage (Timmer and Stone 1978; Figure S1 available as Supplementary data at *Tree Physiology* Online) in the form of amino acids. Under such conditions, growth (Timmer and Stone 1978) and photosynthetic rate (Palmroth et al. 2013, Tarvainen et al. 2016), roughly indicated by foliar δ^13^C, are no longer related to foliar N. Instead, we suggest that assimilation of carbon from the organic N at the early stage may have been responsible for the correlation between biomass production and N concentration, as suggested by Franklin et al. (2017). Using an exponential growth model (Figure 6), a higher growth rate (*r*) was predicted for seedlings on the organic fertilizer (0.050 ± 0.001 *vs* 0.053 ± 0.001, *P* = 0.037). Employing the estimated growth rate, and foliar C:N ratio (35.5 for inorganic *vs* 30.1 for organic fertilizer) together with an assumption that only intact arginine N was taken up at the early stage, seedlings absorbing organic fertilizer would theoretically take up the necessary extra carbon from arginine within 1 week after the onset of the fertilization treatment. Allocating just marginally more carbon, available from arginine uptake, to leaf production may underpin the higher growth rate of seedlings provided with the organic fertilizer. Indeed, ~3% higher relative growth rate would be sufficient to cause the observed ~ 18% higher total biomass by the end of the study (week 10). Thus, even a small increase of relative growth rate, when commencing at an early stage of development, can result in higher biomass without changing physiological traits, especially if the plants reinvest the increased production in increasing foliage surface area (Figure 3).

Studies on theoretical models and observations have highlighted important interactions between N status and water for forest nutrition and production (Tinker and Nye 2000, Franklin et al. 2012, Kulmatiski et al. 2017, Lim et al. 2017, McMurtrie and Näsholm 2018, Sigala et al. 2020, Henriksson et al. 2021). In the current study, we attempted to link root N acquisition and N source with transpiration-relevant leaf and root morphology. We conclude that the enhanced seedling nutrition and growth with the organic fertilizer was mainly achieved due to stable supply of N via diffusive fluxes, without affecting transpiration rate or root or leaf morphology, potentially augmented by carbon obtained through organic N uptake. We also suggest that managing plant nutrition based on organic N can be a possible solution to reduce environmental risk, such as N leaching, while enhancing efficiency of N uptake and maintaining plant nutrition and growth, in particular for species adapted to boreal regions, where high soil acidity and lower temperatures impede N mineralization. We note that our scope of inference is limited to *P. sylvestris* seedlings under the greenhouse-pot condition with ample water supply. Future studies with varying water availabilities are needed to advance understanding of interactions of carbon, N and water in plant eco-physiological performance.

## Conflict of interest

Author Torgny Näsholm declares a conflict of interest as he has shares in, and works part time for, the company Arevo AB that develops, produces, and markets arginine-based, organic fertilizers under the trade name arGrow.

## Supplementary Material

Supplementary_Information_Lim_20210906_tpab127
