# Supplementary material for: Organic nitrogen enhances nitrogen nutrition and early growth of Pinus sylvestris seedlings
Source: Tree Physiol. 2021 Sep 28;42(3):513–22. doi: 10.1093/treephys/tpab127 (PMC8919414; doi:10.1093/treephys/tpab127)
Supplement: Supplementary_Information_Lim_20210906_tpab127 [file supplementary_information_lim_20210906_tpab127.docx]

**Supplementary Information**

**Organic nitrogen enhances nitrogen nutrition and early growth of *Pinus sylvestris* seedlings**

^1^Hyungwoo Lim^*^, ^1,2^Sandra Jämtgård, ^3,4^Ram Oren, ^1^Linda Gruffman, ^2^Sabine Kunz, ^1,2^Torgny Näsholm

Affiliations

^1^Department of Forest Ecology & Management, Swedish University of Agricultural Sciences (SLU), SE-901 83 Umeå, Sweden

^2^Department of Forest Genetics and Plant Physiology, SLU, SE-901 83 Umeå, Sweden

^3^Nicholas School of the Environment, Duke University, Durham, NC, 27708-0328, USA

^4^Department of Forest Science, University of Helsinki, FI-00014, Finland

*Corresponding author

Email: hyungwoo.lim@slu.se

Tel: + 46 90 786 84 15

**Table S1. Soil N fluxes (nmol N m^-2^ s^-1^) from the growth medium of seedling-pots supplied with 5 mM N of the inorganic N (ammonium nitrate) or organic (arginine) fertiliser** (mean ± standard error, n = 8 associated with individual seedlings for each treatment). The effects of N source were determined using a two-sample t-test at each time point.

| Time elapsed  (hour) | Total N flux (nmol m^-2^ s^-1^) | | p-value |
| --- | --- | --- | --- |
|  | Inorganic N | Organic N |  |
| –1 | 5.61 (1.23) | 7.64 (1.58) | 0.328 |
| 1 | 123.84 (22.12) | 58.18 (24.69) | 0.068 |
| 2 | 55.88 (24.25) | 70.68 (34.64) | 0.731 |
| 7 | 154.02 (26.11) | 17.56 (3.46) | **0.001** |
| 22 | 94.60 (18.31) | 31.20 (13.48) | **0.015** |
| 30 | 51.47 (15.32) | 28.39 (7.90) | 0.202 |
| 48 | 12.68 (4.57) | 28.94 (17.01) | 0.383 |
|  | Cumulative total N flux (mmol m^-2^) | |  |
| –1 – 1 | 0.40 (0.12) | 0.42 (0.15) | 0.917 |
| 1 – 2 | 0.61 (0.13) | 0.55 (0.15) | 0.767 |
| 2 – 7 | 2.17 (0.48) | 1.70 (0.46) | 0.492 |
| 7 – 22 | 7.65 (1.02) | 4.18 (0.93) | **0.025** |
| 22 – 30 | 9.60 (1.07) | 5.33 (0.99) | 0.011 |
| 30 – 48 | 11.68 (1.18) | 7.04 (1.56) | 0.014 |
| 48 – 71 | 12.52 (1.19) | 8.54 (1.35) | 0.045 |

**Table S2. Traits of *Pinus sylvestris* seedlings grown on ammonium nitrate based inorganic or arginine based organic fertiliser** (mean ± standard error, n = 6 associated with block). Values are estimates of means and standard errors, generated by the linear mixed model (Eq. 1). The effect of foliar N concentration was observed on shoot dry mass. Therefore, we report here normalized estimates of shoot and total dry mass by the mean foliar N concentration for each treatment.

| Seedlings’ traits | Inorganic N | Organic N | organic/inorganic | p-value |
| --- | --- | --- | --- | --- |
| Diameter at stem base (mm) | 1.72 (0.07) | 1.96 (0.07) | 1.14 (0.04) | **0.002** |
| Height (cm) | 11.39 (0.53) | 12.29 (0.53) | 1.08 (0.05) | 0.120 |
| Leaf biomass (g dw) | 0.31 (0.02) | 0.37 (0.02) | 1.18 (0.09) | **0.040** |
| Shoot dry mass (g dw) | 0.41 (0.02) | 0.50 (0.02) | 1.20 (0.08) | **0.017** |
| Root dry mass (g dw) | 0.13 (0.01) | 0.14 (0.01) | 1.09 (0.09) | 0.340 |
| Total dry mass (g dw) | 0.54 (0.03) | 0.64 (0.03) | 1.18 (0.08) | **0.033** |
| Root:shoot in dry mass | 0.30 (0.02) | 0.28 (0.02) | 0.92 (0.05) | 0.154 |
| Unit needle mass (mg per needle) | 2.33 (0.11) | 2.38 (0.11) | 1.02 (0.06) | 0.710 |
| Leaf area per unit leaf mass (cm^2^ g^-1^) | 126.70 (5.55) | 132.22 (5.55) | 1.04 (0.03) | 0.095 |
| Root area per unit root mass (cm^2^ g^-1^) | 196.50 (7.14) | 203.95 (7.14) | 1.04 (0.04) | 0.328 |
| Leaf area (cm^2^) | 39.27 (3.08) | 48.81 (3.08) | 1.24 (0.09) | **0.015** |
| Root area (cm^2^) | 25.17 (2.25) | 28.05 (2.25) | 1.11 (0.09) | 0.221 |
| Root:leaf in area | 0.64 (0.05) | 0.60 (0.05) | 0.93 (0.08) | 0.381 |
| Foliar N concentration (mg N g^-1^ dw) | 14.40 (1.43) | 17.28 (1.43) | 1.20 (0.04) | **<.001** |
| Root N concentration (mg N g^-1^ dw) | 16.68 (0.66) | 17.73 (0.66) | 1.06 (0.04) | 0.165 |
| Foliar N stock (mg N) | 4.37 (0.56) | 6.45 (0.56) | 1.47 (0.11) | **<.001** |
| Total N stock (mg N) | 7.60 (0.82) | 10.63 (0.82) | 1.40 (0.10) | **<.001** |
| N recovery (%) | 22.62 (2.45) | 31.63 (2.45) | 1.40 (0.10) | **<.001** |
| Foliar δ^15^N (‰) | 5.72 (0.34) | 7.28 (0.34) | 1.27 (0.08) | **0.003** |
| Foliar δ^13^C (‰) | –30.77 (0.16) | –30.86 (0.16) | 1.00 (0.01) | 0.614 |
| Root δ^15^N (‰) | 5.08 (0.44) | 6.15 (0.44) | 1.21 (0.11) | 0.057 |
| Root δ^13^C (‰) | –30.16 (0.20) | –30.16 (0.44) | 0.00 (0.00) | 0.993 |

**Fig. S1. Relationship between nitrogen concentration and absolute content of needles for analysis of nitrogen nutrition status (Timmer and Stone 1978).** Grey circles, the inorganic seedlings; black circles, the organic seedlings in each block; triangles are estimates of mean values for each treatment.

**Fig. S2. (a) Photosynthesis rate per unit leaf area normalised by photosynthetic active radiation (PAR) and (b) stomata conductance per unit leaf area normalised by vapor pressure deficit (VPD). (c) Instantaneous water use efficiency (WUE_i_, photosynthesis per unit of transpiration) and (d) intrinsic water use efficiency (WUE_int_, photosynthesis per unit of stomata conductance) normalised by PAR.** Measurements were made using LI-COR 6400XT, under ambient light and CO_2_ condition (400 ppm). Because we did not control the light condition of the measurement cuvette, values are presented based on light-normalization. Gas-exchange measurements were performed a week before a microdialaysis soil N flux monitoring, on the same seedlings.
